# Improving emotion perception in cochlear implant users: insights from machine learning analysis of EEG signals

**DOI:** 10.1186/s12883-024-03616-0

**Published:** 2024-04-08

**Authors:** Sebastien Paquette, Samir Gouin, Alexandre Lehmann

**Affiliations:** 1 Psychology Department, Faculty of Arts and Science, Trent University, Peterborough, ON Canada; 2 Research Institute of the McGill University Health Centre (RI-MUHC), Montreal, QC Canada; 3 Centre for Research On Brain, Language, and Music (CRBLM), International Laboratory for Brain, Music & Sound Research (BRAMS), Psychology Department, University of Montreal, Montreal, QC Canada; 4 Faculty of Medicine and Health Sciences, Department of Otolaryngology-Head and Neck Surgery, McGill University, Montreal, QC Canada

**Keywords:** Cochlear implant, Emotion perception, Machine learning

## Abstract

**Background:**

Although cochlear implants can restore auditory inputs to deafferented auditory cortices, the quality of the sound signal transmitted to the brain is severely degraded, limiting functional outcomes in terms of speech perception and emotion perception. The latter deficit negatively impacts cochlear implant users’ social integration and quality of life; however, emotion perception is not currently part of rehabilitation. Developing rehabilitation programs incorporating emotional cognition requires a deeper understanding of cochlear implant users’ residual emotion perception abilities.

**Methods:**

To identify the neural underpinnings of these residual abilities, we investigated whether machine learning techniques could be used to identify emotion-specific patterns of neural activity in cochlear implant users. Using existing electroencephalography data from 22 cochlear implant users, we employed a random forest classifier to establish if we could model and subsequently predict from participants’ brain responses the auditory emotions (vocal and musical) presented to them.

**Results:**

Our findings suggest that consistent emotion-specific biomarkers exist in cochlear implant users, which could be used to develop effective rehabilitation programs incorporating emotion perception training.

**Conclusions:**

This study highlights the potential of machine learning techniques to improve outcomes for cochlear implant users, particularly in terms of emotion perception.

## Background

Cochlear implants (CIs) can restore auditory inputs to deafferented auditory cortices. However, the quality of the sound signal transmitted to the brain is severely degraded, which can result in limited functional outcomes. While many CI recipients experience improved speech comprehension, even those with good speech outcomes may struggle with perceiving emotions accurately [1, 2]. This deficit negatively impacts socio-professional integration, quality of life [3], and overall emotional development. Therefore, there is a crucial need for rehabilitation programs that incorporate emotion perception training to improve outcomes for CI recipients.

Many CI users exhibit some level of emotional discrimination ability [1, 2, 4]. Hence, the first step in developing emotion rehabilitation training programs is to understand better CI users’ residual emotion perception abilities. Identifying these residual abilities’ neural underpinnings would substantially help support such training programs. Recently [4, 5], electroencephalography (EEG) was used to quantify the neural biomarkers of emotion perception deficits in CI users. While normal-hearing individuals exhibited some emotion-specific neural activity, conventional analyses failed to detect emotional differentiation in CI users, especially in the later portion of the response (600-850 ms). This absence of differences may represent the participant deficit, but it could also be due to a lack of sensitivity from the selected analysis technique. By using machine learning techniques that can provide more sensitivity for EEG analyses (detect smaller variations or patterns in the data), we aim to identify the emotion-specific patterns of neural activity associated with CI users’ residual emotion perception abilities.

## Methods

Here, we used a supervised machine learning method to model (identify emotion-specific patterns of neural activity) and subsequently establish if we can use these patterns to predict the auditory emotion presented to CI participants. In this case, an above-chance prediction (classification) accuracy would support the notion that emotion-specific acoustic cues can be transmitted via the implant and differentiated by CI users’ auditory perceptual system. This project was approved by the Psychology Institutional Review Board at the University of Montréal, and all participants provided informed consent.

### Participants

The study utilized existing data [4] from 22 CI users, whose sociodemographic and clinical characteristics are detailed in Table 1. Throughout the investigation, participants use their implant’s typical daily settings.

**Table 1:** Sociodemographic and clinical characteristics of CI users

CI users’ Sociodemographic and Clinical Characteristics		(*N* = 22)
Chronological Age, mean (SD)		44.0 (15.0)
Female Sex, no. (%)		17 (77.3)
Duration of Profound Deafness, mean years (SD)		16.3 (15.3)
Age at Implantation, mean (Range)		34.7 (1.7–62.7)
^a^Implantation	^b^Unilateral, no. (%)	14 (63.6)
	Bilateral, no. (%)	8 (36.4)
Duration of CI Use, mean years (SD)		9.3 (6.7)
Speech Intelligibility score in Quiet, % score (SD)		68.3 (18.8)

^a^Different manufacturers were represented, but all stimulation strategies were envelope-based

^b^Users with a contralateral hearing aid were asked to remove it during testing

### Stimuli

Forty-eight stimuli, equally divided between two modalities (voice and music) and three emotion types (happy, sad, and neutral), were used for this study. Half of the stimuli were female and male vocal interjections taken from the Montreal Affective Voices (MAV [6]) set with a mean duration of 1.5 (SD: 0.7) sec. The other half were musical excerpts on the clarinet and violin taken from the Musical Emotional Bursts (MEB [7]) set with a mean duration of 1.7 (SD: 0.6) sec. The selected stimuli from each database were the best recognized by pilot CI users.

### Protocol

Participants were set up for EEG recording and instructed to fix their gaze on a cross in front of them to limit ocular motion. The 48 stimuli were repeated 15 times (720 trials in total) and were presented in random order within each block of 48 trials. To ensure participants were paying attention to the task, in 7% of trials, “clicks” (square waves) were added to the stimulus (embedded 200 ms after the onset), and participants were asked to press the spacebar as soon as they detected them. Sounds were sampled at 44.1 kHz with a 16-bit resolution, presented at 70 dB(A) (integrated) via two loudspeakers (Genelec 8040A) located about 1 m away, at a 45° angle on either side of the participant’s head.

### EEG Equipment

Continuous EEG was recorded using 64 electrodes (referred to henceforth as channels) placed on the scalp, according to the International 10/20 system. Signals were sampled at 1024 Hz (ActiveTwo amplifier; BioSemi) and stored for offline analysis using BioSemi ActiView software.

### EEG data pre-processing

The recorded neural activity was pre-processed offline using EEGLAB version 13.6.5b and ERPLAB version 6.1.3. The complete description of all pre-processing steps (i.e., filtering and corrections) is available in the original study [4]. No further pre-processing was required for our re-analysis as the electrodes and epochs affected by signal loss and eye- or CI-related artifacts were already rejected in the previous study. On average, the signal from 4.8 electrodes was removed for CI participants (caused by occasional signal loss or artifact from the implant), and 40 trials (SD = 32) were rejected for CI users. In the final dataset, a minimum of 5 trials (/15) of each stimulus per participant were still available for analysis.

Complementary information about the participants, stimuli, protocol, EEG equipment, and data pre-processing can be found in the original study [4].

### Data used for classification

Emotion classification was performed on neural activity (µV levels) recorded for each time point (588 features) between the auditory stimuli onset and 2300 ms at CZ (central electrode on the sagittal plane; 10/20 system). The frontocentral region was selected because that is where peak levels of evoked activity were observed in the original study; the Cz electrode was chosen because it necessitated the least data interpolation in our participants. Cz recordings were available in 20 CI users; for the other two participants, Cz data were interpolated from lateral recordings in the C1 and C2 electrodes.

### Emotion prediction

We used a supervised machine learning method (Random Forest classifier [8, 9]) to predict, from each participant’s neural activity, the auditory emotions (happy, sad, neutral) presented to them. This algorithm uses multiple decision trees (sets of rules) on subsets of data to generate the most accurate predictions.

Using repeated stratified 5-fold cross-validation to maintain the same percentage of samples per category (emotions), we partitioned the data into training sets (used for modelling) and test sets (used for predictions). The cross-validation was repeated three times, each with a train-test split of 4:1. The classifier performance (prediction accuracy) was recorded for each repetition and averaged together.

Furthermore, because previous reports [2, 10] suggested a response bias towards positive emotions by CI participants, binary classifications (happy vs. sad, happy vs. neutral, sad vs. neutral) were also conducted to assess whether the algorithm equally predicts all three emotions or neglects one.

### Hyperparameter selection

Data from two randomly selected CI users were used to optimize the algorithm parameters. We tested a variety of hyperparameter combinations through a grid search. Our algorithm default values (specifically, the number of decision trees set to ‘100’ and max features set at ‘sqrt’—the square root of the total number of features) produced the highest accuracies for all classification schemes. Data from these two CI users were excluded from our main analyses; only the prediction accuracies from the remaining 20 participants are presented in the result section.

### Statistical significance determination

We used random permutation tests [11] to provide statistical significance levels (*p*-values) for the decoding accuracies. We performed 200 random permutations of the labels (emotion classes) in the data (within subjects) and computed the classification accuracy for each permutation; by doing so, we established an empirical null distribution of our algorithm classification accuracies on random observations. The tail of the group average distribution was then used to determine a significance boundary for a given rate of tolerated correct classifications that occur by chance (false positives).

If a classification accuracy (from non-permuted labels) is higher than the 95th percentile of this empirical null distribution, it can be reported as significantly different (*p* < 0.05) from chance.

## Results

**Fig. 1:**
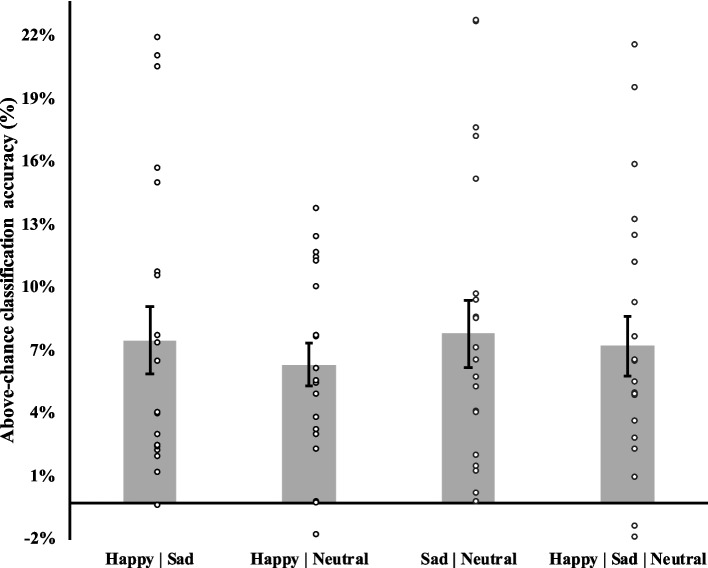
Percent above-chance average (*n* = 20) classification accuracy for the binary (first three bars) and multi-class (fourth bar) analyses. Circles represent individual participants’ classification accuracy, and error bars represent standard error

The classifier algorithm was able to identify emotion-specific neural activity in CI users. It achieved a significant (*p* < 0.05) above-chance emotion classification accuracy for the multi-class problem (identifying emotions from three categories: happy, sad, and neutral; 7.5% above-chance) and for all binary classification (two categories/emotions; Happy-Sad: 7.8%; Happy-Neutral: 6.6%; Sad-Neutral: 8.1%), see Fig. 1 for individual classification accuracy and standard error.

## Discussion

Our study used highly sensitive automated pattern classification to identify emotion-specific patterns of neural activity in CI users. These patterns had previously only been observed in normal-hearing individuals [4].

Prior investigations into the neural correlates of emotion perception in CI users primarily reported that their early evoked responses, such as the N100 and P200, were often attenuated and prolonged compared to controls [4, 5], suggesting a general reduction in the neural encoding of acoustical characteristics transmitted by the implant.

However, in the previously published analysis of the EEG data presented here [4], it was also observed that sad stimuli elicited delayed latencies for both N100 and P200 compared to happy or neutral stimuli in both normal-hearing listeners and CI users. This main effect observed in both groups did not specifically contribute to explaining CI users’ emotion perception deficit. Nonetheless, in the later portion of the evoked response (between 600 and 850), both emotional stimuli (i.e., happy and sad) could be differentiated from the neutral ones in normal-hearing individuals but not in CI users. Since later components of the evoked responses seem to vary with affective content systematically [12–16], this difference between groups was identified as a representation of CI users’ emotion perception deficit.

To uncover the neural basis of residual emotional abilities in CI users (rather than their deficit), we opted for a more comprehensive approach. We analyzed the entire duration of evoked responses and employed a method with heightened sensitivity (i.e., random forest classifier). Through this approach, we successfully identified emotion-specific patterns of neural activity in CI users.

Unlike previous behavioural studies that reported biases towards positive emotion [2, 10] (or difficulties in discerning sadness [2, 4]), the algorithm seems to be able to distinguish all emotions, indicating the presence of consistent emotion-specific biomarkers.

That said, classification schemes involving sad stimuli yielded slightly higher (1.2/1.5%) average classification, potentially reflecting delayed latencies observed for sad stimuli in the original study [4]. Nevertheless, above-chance emotion classification accuracies for all classification schemes support the notion that emotion-specific acoustic cues are transmitted via the implant (e.g., timbre: [1, 17]) to the perceptual system and are available to CI users.

These findings hold significant implications for rehabilitation strategies for CI users. By leveraging these identified biomarkers, tailored training programs [18] could be developed to enhance emotional perception among CI users, potentially leading to an improved quality of life [3].

## Data Availability

All data generated or analysed during this study are included in this published article Deroche MLD, Felezeu M, Paquette S, Zeitouni A, Lehmann A. Neurophysiological Differences in Emotional Processing by Cochlear Implant Users, Extending Beyond the Realm of Speech. Ear Hear. 2019;40:1197–209.
